# Uncommon alliance between MOGAD and Behçet's disease

**DOI:** 10.1093/omcr/omaf165

**Published:** 2025-09-28

**Authors:** Asmae Sikkal, Maha Abdallaoui, Salma Bellakhdar, Hajar Khattab, Kamal Haddouali, E L Moutawakil Bouchra, Mohammed Abdoh Rafai, E L Otmani Hicham

**Affiliations:** Service de neurologie explorations neurophysiologiques clinique, centre hospitalo universitaire Ibn Rochd, Casablanca, 20000, Maroc; Service de neurologie explorations neurophysiologiques clinique, centre hospitalo universitaire Ibn Rochd, Casablanca, 20000, Maroc; Service de neurologie explorations neurophysiologiques clinique, centre hospitalo universitaire Ibn Rochd, Casablanca, 20000, Maroc; Laboratoire génétique et de pathologie moléculaire, faculté de médecine et de pharmacie, université Hassan II, Casablanca, 20000, Maroc; Laboratoire de recherche sur les maladies du système nerveux, neurosensorielles et handicap, faculté de Médecine et de pharmacie, université hassan II, Casablanca, 20000, Maroc; Service de neurologie explorations neurophysiologiques clinique, centre hospitalo universitaire Ibn Rochd, Casablanca, 20000, Maroc; Service de neurologie explorations neurophysiologiques clinique, centre hospitalo universitaire Ibn Rochd, Casablanca, 20000, Maroc; Service de neurologie explorations neurophysiologiques clinique, centre hospitalo universitaire Ibn Rochd, Casablanca, 20000, Maroc; Laboratoire génétique et de pathologie moléculaire, faculté de médecine et de pharmacie, université Hassan II, Casablanca, 20000, Maroc; Service de neurologie explorations neurophysiologiques clinique, centre hospitalo universitaire Ibn Rochd, Casablanca, 20000, Maroc; Laboratoire de recherche sur les maladies du système nerveux, neurosensorielles et handicap, faculté de Médecine et de pharmacie, université hassan II, Casablanca, 20000, Maroc; Service de neurologie explorations neurophysiologiques clinique, centre hospitalo universitaire Ibn Rochd, Casablanca, 20000, Maroc; Laboratoire génétique et de pathologie moléculaire, faculté de médecine et de pharmacie, université Hassan II, Casablanca, 20000, Maroc

**Keywords:** myelin oligodendrocyte glycoprotein antibody associated disease (MOGAD), Behçet's disease, autoimmune disease, recurrence

## Abstract

Myelin oligodendrocyte glycoprotein antibody-associated disease (MOGAD) is a newly recognized demyelinating disease with diverse clinical and radiological manifestations that continue to expand over time. Its coexistence with Behçet's disease, a multisystem inflammatory disorder, has not been previously reported. We report a rare case of a 40-year-old female with Behçet's disease who was treated with colchicine and developed two attacks of longitudinal extensive transverse myelitis, associated with positive serum anti-MOG antibodies. During the first episode, the patient responded well to five days of intravenous methylprednisolone associated with two plasma exchange sessions. Although recommended, the patient declined Mycophenolate Mofetil treatment until the second episode. Since then, she has remained free of relapses during two years of follow-up, reinforcing the hypothesis that a background of autoimmune dysregulation may predispose patients to MOGAD and lead to its recurrence.

## Background

Myelin oligodendrocyte glycoprotein antibody-associated disease (MOGAD) is becoming more widely acknowledged as a distinct illness that exhibits a wide spectrum of clinical manifestations, including encephalitis and demyelinating disorders. MOGAD can have a monophasic or a recurrent course. Over 50% of patients experience relapses [1], which can be attributed to a variety of unidentified factors, such as markers in the cerebrospinal fluid (CSF), antibody titers, and clinical features. It has also been proposed that past immunological conditions can serve as catalysts for relapse.

We report a case of MOGAD recurring with longitudinally extensive transverse myelitis (LETM), which occurred in Behçet's disease. To our knowledge, this is the first case in the literature to report this presumed relationship.

## Case presentation

**Figure 1:**
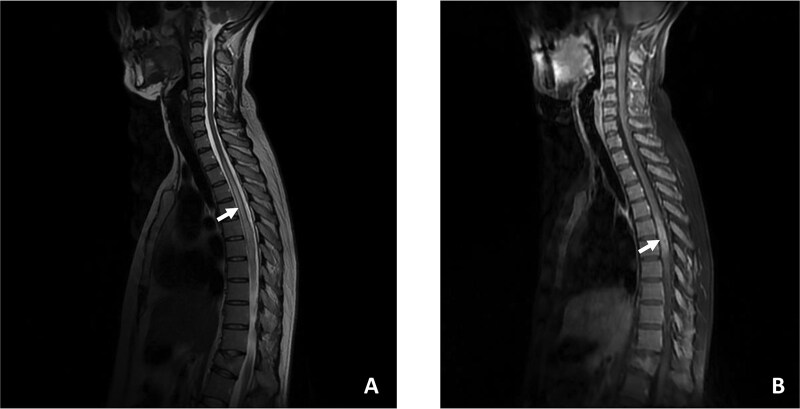
T2-weighted magnetic resonance image showing an extensive hyperintensity from D1 to D8 (A), with gadolinium enhancement (B).

**Figure 2:**
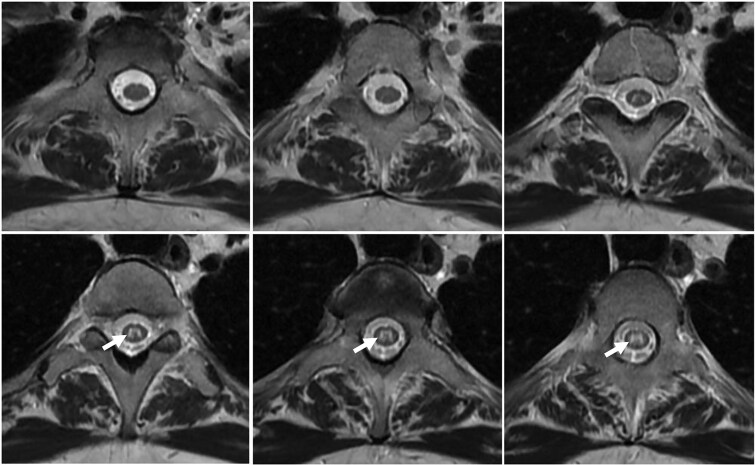
Axial T2-weighted magnetic resonance image showing the ‘H sign’.

A 40-year-old female diagnosed with Behçet's disease since 2014 (characterized by five annual episodes of bipolar aphthosis, pseudofolliculitis skin involvement and inflammatory arthralgia with positive HLA B51) and treated with colchicine, presented with acute progressive weakness in the lower extremities and voiding dysfunction for over three days. On neurological examination, the patient was bedridden, presenting with asymmetric paraplegia and sensory level at D4. The Babinski reflexes were positive on both sides. T2-weighted magnetic resonance imaging (MRI) revealed an extensive hyperintensity from D1 to D8, with gadolinium enhancement (Fig. 1), and displayed an ‘H sign’ in axial sequences (Fig. 2)*.* Brain MRI were normal. CSF analysis revealed a white blood cell count of 10/mm3 and normal protein and glucose levels, with negative oligoclonal bands. Routine CSF culture and Gram-stained samples were negative. Serum anti-myelin oligodendrocyte glycoprotein (MOG) antibodies were positive (titer of ≥1:100), as confirmed by a live cell-based assay (CBA). Anti-aquaporin-4 (AQP4) antibodies and other systemic autoimmune markers, including antinuclear antibodies (ANA), anti–double-stranded DNA (anti-dsDNA) antibodies, antiphospholipid antibodies, and anti-SSA/SSB antibodies, were all negative.

The patient was treated with of intravenous methylprednisolone (1000 mg/day) for five days and underwent two plasma exchange sessions. There was a significant improvement in mobility, with the patient regaining the ability to walk without assistance within three weeks. A tapered course of oral steroids was started, and although recommended, the patient initially declined Mycophenolate Mofetil (MMF) treatment.

One year later, and precisely 15 days after completing the steroid taper, a similar relapse occurred, rendering the patient bedridden. Laboratory tests revealed the presence of anti-MOG antibodies (CBA test). After partial improvement following another course of corticosteroid infusion, long-term treatment with MMF was initiated, leading to no neurological relapses during the two-years follow-up. The patient maintained mobility but required some assistance to walk (mRS of 2).

## Discussion

In this report, we describe a case that illustrates the cooccurring manifestations of two autoimmune disorders: Behçet's disease, diagnosed according to international criteria, and MOGAD, as evidenced by positive serum anti-MOG antibodies and LETM marked by a distinctive ‘H sign’, a feature more typically associated with MOGAD [2] than with the myelitis seen in Behçet's disease which instead presents with the ‘Bagle sign’ [3]. Notably, while MOGAD can present with symptoms similar to those of neuro-Behçet's disease [4], several elements argue against the latter in our case: the long-standing remission under colchicine for nearly 10 years (one of the trailblazer treatments in BS), absence of CNS parenchymal lesions (brainstem lesions), and MRI features atypical for Behçet’s disease. Moreover, Colchicine, used by the patient for Behçet’s disease, exerts anti-inflammatory effects via inhibition of the NLRP3 inflammasome and IL-1β signalling [5]. However, these innate immune mechanisms are likely insufficient to prevent CNS demyelination in MOGAD, which involves distinct autoantibody-mediated pathways.

This case supports the hypothesis that a background of immune dysregulation (Behçet’s disease) may predispose individuals to MOGAD and contribute to its recurrence, reinforcing the need for the early implementation of disease-modifying treatments. Recent evidence supports the early initiation of long-term immunosuppressive therapy in patients at higher risk of relapse, such as comorbid autoimmune diseases [1, 6]. One report from China revealed that approximately one-third of patients with MOGAD had a history of other immunological conditions, a factor significantly linked to the occurrence of relapses [1]. In contrast, the simultaneous presence of autoimmune diseases appears to be less common in MOGAD than in Neuromyelitis Optica Spectrum Disorder (NMOSD) [7], indicating that different immunopathogenic mechanisms may govern each condition.

## Conclusion

This case highlights that demyelinating syndromes occurring in patients with known autoimmune conditions warrant specific investigations, including MOG antibody testing. Although a causal link cannot be established based on a single case, and based on recent studies [1, 6], we illustrate the possibility that underlying immune dysregulation, such as Behçet’s disease, may influence MOGAD’s relapse risk and management strategy. In our case, the positive outcome with MMF after relapse retrospectively supports this approach. This emphasizes the need for individualized long-term management strategies from the first attack in selected patients.

## Consent

Written informed consent was obtained from the patient for publication of the case report and is available for review by the editor of this journal.
